# Transforming the delivery of care from “I” to “We” by developing the crisis resource management skills in pediatric interprofessional teams to handle common emergencies through simulation

**DOI:** 10.1186/s12909-024-05459-2

**Published:** 2024-06-11

**Authors:** Sana Saeed, Nagwa Nashat Hegazy, Marib Ghulam Rasool Malik, Qalab Abbas, Huba Atiq, Muhammad Maisam Ali, Aashir Aslam, Yasmin Hashwani, Farzana Bashir Ahmed

**Affiliations:** 1 Department of Pediatrics and Child Health, and Department of Educational Development, The Aga Khan University, Karachi, Pakistan; 2 Family Medicine Department, Faculty of Medicine, Menoufia University, Menoufia, Egypt; 3 Dean’s Clinical Research Fellowship Program, The Aga Khan University, Karachi, Pakistan; 4 Department of Pediatrics and Child Health, The Aga Khan University, Karachi, Pakistan; 5 Department of Anesthesiology, Department of Emergency Medicine, and Center of Excellence for Trauma and Emergencies, The Aga Khan University, Karachi, Pakistan; 6 Medical College, The Aga Khan University, Karachi, Pakistan

**Keywords:** Crisis Resource Management, Simulation-based training, Interprofessional teams

## Abstract

**Background:**

The healthcare system is highly complex, and adverse events often result from a combination of human factors and system failures, especially in crisis situations. Crisis resource management skills are crucial to optimize team performance and patient outcomes in such situations. Simulation-based training offers a promising approach to developing such skills in a controlled and realistic environment.

**Methods:**

This study employed a mixed-methods (quantitative-qualitative) design and aimed to assess the effectiveness of a simulation-based training workshop in developing crisis resource management skills in pediatric interprofessional teams at a tertiary care hospital. The effectiveness of the intervention was evaluated using Kirkpatrick’s Model, focusing on reaction and learning levels, employing the Collaboration and Satisfaction about Care Decisions scale, Clinical Teamwork Scale, and Ottawa Global Rating Scale for pre- and post-intervention assessments. Focused group discussions were conducted with the participants to explore their experiences and perceptions of the training.

**Results:**

Thirty-nine participants, including medical students, nurses, and residents, participated in the study. Compared to the participants’ pre-workshop performance, significant improvements were observed across all measured teamwork and performance components after the workshop, including improvement in scores in team communication (3.16 ± 1.20 to 7.61 ± 1.0, *p* < 0.001), decision-making (3.50 ± 1.54 to 7.16 ± 1.42, *p* < 0.001), leadership skills (2.50 ± 1.04 to 5.44 ± 0.6, *p* < 0.001), and situation awareness (2.61 ± 1.13 to 5.22 ± 0.80, *p* < 0.001). No significant variations were observed post-intervention among the different teams. Additionally, participants reported high levels of satisfaction, perceived the training to be highly valuable in improving their crisis resource management skills, and emphasized the importance of role allocation and debriefing.

**Conclusions:**

The study underscores the effectiveness of simulation-based training in developing crisis resource management skills in pediatric interprofessional teams. The findings suggest that such training can impact learning transfer to the workplace and ultimately improve patient outcomes. The insights from our study offer additional valuable considerations for the ongoing refinement of simulation-based training programs. There is a need to develop more comprehensive clinical skills evaluation methods to better assess the transferability of these skills in real-world settings. The potential challenges unveiled in our study, such as physical exhaustion during training, must be considered when refining and designing such interventions.

**Supplementary Information:**

The online version contains supplementary material available at 10.1186/s12909-024-05459-2.

## Background

The healthcare system is highly complex, and despite extensive research, modern technology, and rigorous training, adverse events are estimated to occur in approximately 10% of all patients [1]. Adverse events in healthcare are often the result of combinations of human factors and systems failures. In the United States alone, up to 100,000 patients die because of medical errors each year [2]. This problem is magnified in a crisis situation due to the pressure to act quickly with the stress of caring for an unstable patient [3]. Current interventions primarily focus on implementing safety tools such as event-reporting systems, quality and safety dashboards, checklists, and evidence-based guidelines [4]. However, it has been shown that introducing more stringent measures may actually increase the gap between procedure and practice [5]. For instance, while medical checklists are designed with careful consideration of real-world clinical dynamics, their rigid implementation with limited flexibility to adapt to unique patient circumstances, such as high-risk and critically ill patients, and limited consideration of team dynamics may lead to deviations from recommended procedures in favor of more practical or contextually appropriate approaches [6]. To deliver safe and effective care, medical professionals must execute highly coordinated team-based strategies. Inter-professional team-based collaboration is a process in which health professionals from different disciplines work together to deliver high-quality, evidence-based care to ensure optimal patient outcomes [7]. Patient outcomes are highly dependent on coordinated and collaborative efforts of team members from interprofessional and interdisciplinary domains [8]. A recent systematic review and meta-analysis was conducted to assess the effectiveness of interprofessional learning to improve mortality rates and other patient outcomes such as length of hospital stay, achievement of therapeutic outcomes and occurrence of adverse events when compared to conventional care practice [9]. The meta-analyses found a significantly reduced overall risk of mortality (Risk ratio: 0.72 [0.60–0.86]) as well as a significantly reduced overall risk of adverse outcomes (Risk ratio: 0.77 [0.67–0.88]) in the interprofessional learning group compared to the control group. The non-technical skills of a crisis, including collaboration, communication, teamwork, task management, and leadership, are also highly critical to effective patient management [10].

Crisis resource management (CRM) refers to a set of principles defining cognitive and interpersonal behaviors that contribute to optimal team performance and better outcomes [11, 12]. The key principles taught in CRM are designed to facilitate earlier detection of potential adverse outcomes and empower healthcare practitioners to intervene more effectively. Simulation-based training offers a unique opportunity for healthcare teams to practice and assess CRM behaviors, by managing realistic clinical scenarios in a controlled setting without posing a threat to patient safety. It has been utilized in various training programs to improve outcomes and the healthcare delivery system [13]. It can be summarized through eight teamwork behaviors: leadership, communication, anticipation and planning, resource utilization, workload distribution, situational awareness, triage and prioritization, and management of disruptions [2]. Simulation-based training has shown an exponential growth to address issues of patient safety and competencies unique to a given specialty [11]. Literature has suggested that simulation-based CRM training positively impacts the transfer of learning to the workplace and on patient outcomes [12].

The Aga Khan University (AKU) is known internationally for the quality of its training programs, e.g., Bachelor of Medicine and Bachelor of Surgery (MBBS), Bachelor of Nursing, and residency programs. However, these programs involve training the individual within the boundaries of disciplines, with little room for interprofessional learning. There is immense evidence on the importance of multidisciplinary education and its effects on patient-based outcomes [11, 12]. It also encourages learners to perform, reflect and learn the best behaviors in patient management through curriculum innovation and teamwork.

## Methods

This study aimed to evaluate the effect of simulation-based training of interprofessional, team-based learning on acquiring CRM skills among healthcare professionals from multiple disciplines at AKU. It also explored the experiences of the team regarding the training program. The study adopted a mixed-methods (quantitative-qualitative) design and was conducted in the Department of Pediatrics and Child Health at AKU.

### Participants and sampling

Participants were recruited through purposive sampling, targeting a range of healthcare professionals: MBBS Year 4 students, Post-graduate Year 3 residents, and critical care nurses. Any individuals who refused to participate were excluded. Participants were divided into teams of 5–6 individuals. To ensure diverse perspectives, the teams comprised individuals from varied professional roles, with at least one medical student, resident, and nurse in every team. All participants provided written informed consent to participate prior to initiation of the study.

### Training intervention

The core of the training involved a five-hour simulation-based workshop, covering four key pediatric emergencies: shock, respiratory failure, continuous seizures, and trauma. The participants were divided into three teams which were named: “savior”, “protector”, and “rescuer”. These team names were specifically chosen to immerse participants more deeply in the simulation experience and underscore the significance of their roles as lifesavers. The training encompassed both practical simulations and theoretical learning, facilitated through debriefing sessions. Additionally, the workshop included facilitator-led discussions on CRM skills and video-based discussions.

### Quantitative data collection

In order to evaluate the effectiveness of the intervention, this study employed Kirkpatrick’s Model, an established framework for assessing training program outcomes [14]. Kirkpatrick’s Model encompasses four levels of evaluation: Reaction, Learning, Behavior, and Results. The effectiveness of this project was evaluated by specifically focusing on the first two levels of the model. In Level 1 – ‘Reaction’ – participants’ feedback regarding the structure, content, and duration of the training was obtained using a pre-structured tool. This tool was based on a 5-point Likert scale (1: Strongly disagree, 5: Strongly Agree) and comprised of 10 items. For Level 2 – ‘Learning’ – the assessment of participants’ performances was conducted before and after the intervention using a triangulated approach, incorporating three distinct yet complementary validated evaluation tools. The Participants’ Team Collaboration and Satisfaction about Care Decisions (CSACD) was utilized to gauge the effectiveness of team dynamics and decision-making within healthcare settings. This tool helped in understanding how well the teams worked together and their satisfaction with the care decisions made [15]. Additionally, the Clinical Teamwork Scale (CTS) was employed to specifically evaluate clinical teamwork, focusing on critical aspects like effective communication, role clarity, decision-making, and situational awareness [16]. The CTS is designed to be utilized by raters with minimal training. Lastly, the Ottawa CRM Global Rating Scale (GRS) was used to assess the broader competencies, offering a holistic assessment of the participants’ abilities to manage situations effectively [17]. The Ottawa GRS employs a seven-point Likert scale and assesses the performance of simulation CRM scenarios based on five aspects: leadership skills, problem solving, resource utilization, situation awareness, and communication skills. The tool includes rating guidance for most response choices and can be easily applied by an expert evaluator [17, 18].

### Rating process

The performance evaluation of the teams was carried out by two independent evaluators proficient in intensive care, both identified as experienced Intensivists with over 5 years of intensive care practice. Before the simulation training, the evaluators underwent a one-hour rater training session on the scope and usage of the Ottawa GRS. Subsequently, during the actual simulation activities, both evaluators observed and assessed all team performances in real-time and on-site using the CTS and Ottawa GRS. Each evaluator rated each performance on both scales. Throughout the evaluation process, the evaluators maintained independence by not discussing the performances with each other and by scoring each team’s performance on separate evaluation sheets, ensuring unbiased and objective assessments of the teams’ competencies. Since the same evaluators were involved in the rating of the performances pre- and post-training, we were unable to blind the raters regarding the timing of each performance relative to the training intervention as they automatically knew when they were rating pre-training or post-training performances.

### Qualitative data collection

The qualitative arm of the study involved participants engaging in focused group discussions (FGDs) post-workshop. A total of 3 FGDs were conducted, with 6–8 participants per discussion who were recruited via purposive sampling and provided written informed consent to participate. The discussions were conducted with each level of learners i.e., medical students, postgraduate trainees, and nurses, to obtain a maximum variation of responses. The FGDs explored the participants’ prior experiences in CRM situations and training, their experience of the current simulation-based training, including its strengths, challenges, and future recommendations to facilitate an effective learning experience. The interview guide of the FGD can be found in the Supplementary Material of the manuscript (Supplementary Material 1). The discussion were audio recorded, and recordings were then transcribed to obtain verbatim quotes from the participants.

### Quantitative analysis

Quantitative data were analyzed using statistical software for data science (STATA) version 15.1 [19]. Demographic details of the participants were presented as frequencies and percentages, while scores for each of the items of the training evaluation questionnaire and the three performance-assessment scales were expressed as means ± standard deviations. Paired t-tests were applied to CSACD scores reported by the participants pre- and post-intervention, to assess their satisfaction with the training. Evaluator ratings of each item of the CTS and Ottawa GRS from both evaluators were averaged for each performance to obtain the final mean rater scores. Paired t-tests were applied to compare the mean rater scores pre- and post-training. Bonferroni corrections were applied for multiple comparisons to obtain adjusted alpha values for each scale.

### Qualitative analysis

Transcribed data from the FGDs underwent thematic analysis. Analysis was conducted to identify patterns in the participants’ responses and exploring note-worthy aspects that may form the basis of themes across the dataset. The themes were assigned unique codes through deductive reasoning and relevant quotes from each discussion were added to the specific code [20]. Similar categories within a theme were clumped together to yield subthemes. The analysis was conducted independently by two researchers. NVivo software was used to facilitate the organization and management of the qualitative data [21].

## Results

### Quantitative results

The quantitative arm of the study included a total of 39 participants, of whom 46% were medical students (*n* = 18), 28% nurses (*n* = 11), and 26% residents (*n* = 10). 54% identified as male (*n* = 21), and 46% as female (*n* = 18), with the average age being 25. The vast majority of participants (87%, *n* = 34) reported no prior experience in CRM. The participants’ evaluation of the workshop is presented in Table 1 and showed high levels of satisfaction with the workshop’s content, importance, and relevance.

**Table 1:** Participants’ evaluation of the workshop*

Characteristics	Rating (Mean ± SD)
I feel confident in understanding the concept of Crisis Resource Management Skills.	4.15 ± 0.99
I feel confident in demonstrating my role during Crisis Resource Management Skills.	4.05 ± 1.00
The training was appropriate according to my level of understanding.	4.05 ± 1.07
The training stimulated and challenged my critical thinking.	4.23 ± 1.04
The workshop effectively utilized simulation for training on Crisis Resource Management Skills.	4.31 ± 1.08
The team created a safe learning environment where I learned without fearing being judged on my capacity.	4.31 ± 1.08
The post-simulation debriefing helped me in learning CRM skills at a deeper level.	4.31 ± 1.10
The trainers were skilled and facilitated my learning of CRM skills.	4.49 ± 0.85
The trainers gave me enough time to clarify my queries.	4.26 ± 1.07
I would like to attend such training workshops in future	4.38 ± 1.09

*5-point Likert scale (1 = strongly disagree, 5 = strongly agree)

To evaluate the effect of the intervention on team collaboration and satisfaction about care decisions, the CSACD tool was employed. The intervention significantly improved the participants’ team collaboration and satisfaction about care decisions, as demonstrated in Table 2. The mean pre-intervention scores for each item ranged from 4.82 to 5.77, while the mean post-intervention scores ranged from 6.37 to 6.6. All differences were significant (*p* < 0.001). For example, participants reported a significant improvement in planning together to make decisions about patient care (pre: 5.45 ± 0.14, post: 6.45 ± 0.08), as well as satisfaction with the decision-making process (pre: 5.17 ± 0.15, post: 6.37 ± 0.09) and the decision itself (pre: 4.82 ± 0.22, post: 6.52 ± 0.08). Furthermore, there were no significant differences by team or occupation.

**Table 2:** Pre-Post differences in Participants’ Team Collaboration and Satisfaction about Care Decisions (CSACD)*

Items	Pre-intervention mean ± SD	Post-intervention mean ± SD	*p*-value**
Team members planned together to make the decision about care for this patient.	5.45 ± 0.14	6.45 ± 0.08	**< 0.001**
Open communication among team members took place as the decision was made for this patient.	5.27 ± 0.13	6.42 ± 0.09	**< 0.001**
Decision-making responsibilities for this patient were shared among team members	4.95 ± 0.20	6.60 ± 0.07	**< 0.001**
Team members cooperated in making the decision	5.5 ± 0.17	6.47 ± 0.08	**< 0.001**
In making the decision, all team members’ concerns about this patient’s need were considered	5.77 ± 0.14	6.47 ± 0.10	**< 0.001**
Decision-making for this patient was coordinated among team members.	5.12 ± 0.19	6.47 ± 0.07	**< 0.001**
How much collaboration among team members occurred in making the decision for this patient?	5.27 ± 0.15	6.47 ± 0.10	**< 0.001**
How satisfied are you with the way the decision was made for this patient, that is with the decision-making process, not necessarily with the decision itself?	5.17 ± 0.15	6.37 ± 0.09	**< 0.001**
How satisfied were you with the decision made for this patient?	4.82 ± 0.22	6.52 ± 0.08	**< 0.001**

*7-point Likert scale (1 = strongly disagree, 7 = strongly agree)

**Adjusted alpha = 0.006

The intervention also positively affected all overall measures of performance per the CTS. Overall teamwork and team communication scores increased from 3.16 ± 1.20 to 7.61 ± 1.09 and 3.16 ± 1.24 to 7.61 ± 1.28, respectively (*p* < 0.001). Overall situational awareness rating significantly improved from 2.66 ± 2.05 to 7.27 ± 1.48, decision-making rating rose from 3.50 ± 1.54 to 7.16 ± 1.42, while overall role responsibility rating improved from 2.50 ± 1.54 to 8.66 ± 1.02 (*p* < 0.001) (Table 3). Of note, none of the measures reflected any statistically significant variations post-intervention across the three teams.

**Table 3:** The difference in the performances of teams as measured by Clinical Teamwork Scale (Mean Rater Scores)*

Items	Pre-intervention mean ± SD	Post-intervention mean ± SD	*p*-value**
**Overall Teamwork**	3.16 ± 1.20	7.61 ± 1.09	**< 0.001**
**Overall Team Communication**	3.16 ± 1.24	7.61 ± 1.28	**< 0.001**
1. Orient new members (SBAR)	4.22 ± 2.12	7.16 ± 1.15	**< 0.001**
2. Transparent thinking	3.66 ± 1.74	7.27 ± 1.44	**< 0.001**
3. Directed communication	3.66 ± 2.19	7.94 ± 1.30	**< 0.001**
4. Closed loop communication	1.94 ± 1.83	7.55 ± 1.58	**< 0.001**
**Overall Situational Awareness Rating**:	2.66 ± 2.05	7.27 ± 1.48	**< 0.001**
1. Resource allocation	2.55 ± 2.22	7.38 ± 1.33	**< 0.001**
2. Target fixation	1.33 ± 0.48	1.72 ± 0.46	0.0151
**Overall Decision-making Rating**:	3.50 ± 1.54	7.16 ± 1.42	**< 0.001**
1. Prioritize	3.38 ± 1.75	7.00 ± 1.28	**< 0.001**
**Overall Role Responsibility (Leader/Helper) Rating**:	2.50 ± 1.54	8.66 ± 1.02	**< 0.001**
1. Role clarity	2.33 ± 2.16	8.44 ± 1.04	**< 0.001**
2. Perform as a leader	2.38 ± 2.06	8.33 ± 1.13	**< 0.001**
3. Perform as a helper	2.38 ± 2.06	8.44 ± 1.24	**< 0.001**
**Patient Friendly**:	4.16 ± 1.75	8.44 ± 0.85	**< 0.001**

*10-point scale (0 = not acceptable, 10 = perfect)

**Adjusted alpha = 0.003

SBAR: Situation, Background, Assessment, and Recommendation

The intervention was successful in significantly improving all skills assessed by the Ottawa GRS. The greatest raises were observed in the communication skills (pre: 5.55 ± 0.78; post: 2.50 ± 1.29, *p* < 0.001) and leadership (pre: 5.44 ± 0.61; post: 2.50 ± 1.04, *p* < 0.001) (Table 4).

**Table 4:** The difference in the performances of teams as measured by Ottawa Global Rating Scale (Mean Rater scores)*

Items	Pre-intervention mean ± SD	Post-intervention mean ± SD	*p*-value**
Leadership skills	2.50 ± 1.04	5.44 ± 0.61	**< 0.001**
Problem solving skills	3.16 ± 1.46	5.66 ± 0.90	**< 0.001**
Resource utilization	2.61 ± 1.13	5.33 ± 0.90	**< 0.001**
Situational Awareness	2.61 ± 1.13	5.22 ± 0.80	**< 0.001**
Communication skill	2.50 ± 1.29	5.55 ± 0.78	**< 0.001**
Overall	3.44 ± 0.92	6.00 ± 0.76	**< 0.001**

*(7-point scale: 1 = novice, 7 = clearly excel)

** Adjusted alpha = 0.008

### Qualitative results

The qualitative arm of the study explored participants’ experiences and perceptions regarding interprofessional experiential learning CRM skills. Several unique themes and subthemes regarding the strengths and challenges of the simulation training were derived from the FGDs with the participants (Table 5).

### Experiential learning

Participants shared their experiences of learning CRM through interprofessional training. Although certain participants had prior experience of CRM from real-life clinical scenarios, this was the first time they engaged in this in a simulated, collaborative environment. They acknowledged the different focus of the workshop, shifting from individual patient management to a more holistic approach. Their training also taught them to maintain composure, ensuring clear communication within the team to avoid panic and negatively impacting patient outcomes.

*I have observed such situations in my clinical setting, and I have noticed in real-life we are more panicked, but today we learned how to be composed and calm to manage the patient to have clear communication within our team, and there shouldn’t be any chaos because that can affect patient outcomes*.

Participants reported satisfaction with the training, noting significant improvements in their performances through better teamwork and resource management. They mentioned the workshop helped them understand the importance of assigning roles, managing the situation calmly, and avoiding panic, leading to improved prognosis. The randomized team assignment during the workshop replicated real-life situations, enabling participants to experience authentic challenges and improve their skills.

*When we are trying to save a patient, we don’t look at other things, like if they’re organized or not. Here, we saw it from a different perspective, and I feel we learned to stick to roles and manage resources, which I feel lacked in other situations I have been in*.

Participants appreciated the feedback provided during the session, highlighting strengths and weaknesses, which allowed them to identify areas for improvement. The experience helped them learn crucial team dynamics, leadership qualities, communication skills, and the ability to trust their team members, preventing emotions from influencing critical decision-making and promoting efficiency to prevent chaos during a crisis situation. At the same time, it was suggested to hold multiple simulations simultaneous to streamline the overall process.

Participants considered the post-performance debriefing critical to learning; receiving feedback allowed them to understand errors and improve accordingly. They appreciated the workshop’s interactive nature, which facilitated practical learning and made it engaging and enjoyable. The hands-on, scenario-based approach was particularly valued, as it simulated real-life patient care situations and allowed participants to gain practical experience.

### Participants’ recommendations for future sessions

Participants expressed a preference for conducting sessions on days without other obligations, for an undistracted and focused environment.

I really don’t want to stay back till 5PM if it can be avoided. If this workshop is to be done, can we not have anything else on the same day because it is pretty exhausting.

While the feedback provided focused on team dynamics, participants also recommended evaluating clinical skills to enhance overall performance. To further improve learning, participants recommended a briefing of the workshop’s objectives, to be better prepared prior to sessions.

At the same time, they advocated for incorporating such sessions as part of the standard curriculum early in medical school, to provide students valuable skills and confidence at an initial stage in their career. They recommended incorporating the sessions into various rotations, irrespective of the specialty, and frequently, to comprehensively cover all aspects of patient care. They emphasized the importance of interprofessional training to promote diverse perspectives during real-life situations.

*“I think if such simulations are conducted frequently, where consultants, residents, and students can be in the same, then every member of the team might be more comfortable in giving constructive feedback when a real situation is happening.”*

Participants recommended that such workshops should be included in the longitudinal curricula of students, trainees as well as physicians with more frequent opportunities to communicate with each other, which would allow all the participants to be able to receive and provide feedback more comfortably.

### Integration into practice

Participants showed optimism toward integrating experiential, interprofessional learning into real-life clinical scenarios. The workshop’s focus on role allocation, closed-loop communication, and teamwork was seen as crucial in reducing errors and improving care. Additionally, participants appreciated that new knowledge could be shared by any team member, improving the learning curve for everyone.

*It will help in reducing errors and improving patient outcomes, and also teach how to behave with people of different programs and work cohesively*.

Overall, the participants showed great satisfaction with the training and displayed confidence in its potential to improve their individual and interpersonal skills, and in turn improve healthcare outcomes.

I think we will see results and improvements in all areas, so patient care and interpersonal relationships will benefit.

**Table 5:** Themes, subthemes and verbatim quotes from focused group discussions regarding the training

Theme	Subtheme	Quote
**Experiential learning**	Simulated environment	*“You always remember more when you work practically, rather than a presentation. Especially as a resident, you are expected to deal with them, so hands-on I feel was the best part.”*
	Holistic approach	*“Previously, we were more focused on managing the patient. Today, we were focused on managing the crisis situation, assigning roles, good communication, and efficiency.”*
	Team dynamics	*“During this stimulation process, I learned about how you trust your team and how to not let your emotions affect the situation.”*
	Feedback	*“I liked the fact that we got to learn from each other; moreover, the pre- and post-assessment really helped us find our weaknesses and strengths.”*
**Participants’ recommendations for future sessions**	Early incorporation in training	“*I feel that this whole activity can be a part of our normal curriculum, starting with the students at the longitudinal theme level, then for the staff and residents”*
	Inclusion of other specialties	*“It can be a part of their skills based on their rotation, and of any rotation, not just Pediatrics*.”
	Frequent sessions	*“I think that we should conduct such workshops more frequently and have more practice. All aspects of care should be taught more. I suggest offering it as part of blended program to save our time and learn it holistically.”*
**Integration into practice**	Interprofessional learning	*“There is a hierarchy you do reach in patient management, but this workshop showed that students, residents, and even fellows were on the same level. Everyone knew something which affected patient management daily, and that’s where knowledge-sharing really helps.”*
	Enhanced preparedness	*“This training was productive, because if I face such a situation in real life, I will be ready, and I will also be able to teach my colleagues.”*
	Improved outcomes	*“It will help in reducing errors and improving patient outcomes, and also teach how to behave with people of different programs and work cohesively.”*

## Discussion

The current study aimed to evaluate the effectiveness of interprofessional, simulation-based training in developing CRM skills among healthcare professionals. Our results portray this approach to training to serve as a highly effective method for enhancing the various critical dimensions of CRM which are vital for the effective management of emergency and crises situations in healthcare settings. Our findings emphasize the transformative potential of these skills in pediatric interprofessional teams, particularly when honed through simulation-based training.

Interprofessional care is a core value of healthcare, and strongly linked to patient safety and outcomes [9, 22]. The overall structure of the intervention was appreciated by participants, noting the simulated environment allowed them to practice real-life scenarios using a multidisciplinary and collaborative approach. Similarly, Wu et al. reported CRM training was perceived to improve team behaviors, reduce errors, and enhance overall care [23].

The study showed CRM training significantly improved multiple performance predictors, including prioritization, situational awareness, shared responsibility, and quality of leadership. Participants learned how role allocation and appropriate resource management influenced critical decision-making and prognosis. At the same time, they appreciated new knowledge could be shared by any member, improving the learning curve. Alegret et al. highlight this allows each team member to feel integrated, clearly understand their function, and participate at all times in care [24].

In the qualitative arm of our study, participants offered insightful reflections on their CRM training experiences. They described a shift in their focus from individual patient management to a more holistic approach, emphasizing the importance of clear communication and composure during crisis situations. Participants also cited the post-performance debriefing as important for learning, as they were able to note their strengths and weaknesses. Boet el al as well as LeFlore and Anderson both underscored the importance of the facilitator’s role providing constructive feedback, as this led to superior outcomes compared to self-directed learning [25, 26].

While participants expressed the benefits of CRM training, they also highlighted its potential challenges, such as physical exhaustion and the need for more comprehensive clinical skill evaluation. A targeted systematic review by Hippe et al. noted the importance of understanding the costs, processes, and outcomes to ensure the long-term viability and effectiveness of simulation-based education [27]. The insights from our study offer additional valuable considerations for the ongoing refinement of simulation-based training programs.

Not all studies demonstrated a clear benefit of CRM training: Hicks et al., Clarke et al., and Parsons et al. all failed to show a statistically significant difference using the GRS [28–30]. However, each of these studies included a cohort of emergency medicine trainees, not an interprofessional team, and prior experience may have been a confounder. In contrast, the majority of participants in this study reported no exposure to CRM. This was the first study conducted in the Pakistani population, and participants suggested incorporating CRM principles into the standard curriculum early during medical school, tailored to individual rotations, to provide students these skills in the initial phase of their career.

One of the key strengths of our study is the comprehensive approach to evaluating CRM training in pediatric interprofessional teams, capturing both quantitative and qualitative data. Furthermore, the use of validated scales lends credibility to the findings. However, the study is not without its limitations. First, the self-reported improvements in skills like teamwork and communication could be influenced by social desirability bias. Additionally, the limited sample size and the focus on a single institution may affect the generalizability of our findings. An important limitation pertains to the inability of blinding during the performance rating process by the evaluators, where both evaluators in our study were aware of whether the performance they were rating was pre- or post-intervention, introducing a risk of rater bias. Future studies should aim to minimize this bias by perhaps providing video recordings of participant performances to the evaluators so as to blind them with regards to the timing of the performances in relation to the intervention. Lastly, the study focused its clinical scenarios to a single discipline, which may not capture the full range of challenges and benefits associated with CRM training. Future research is recommended to validate these findings in a broader population.

## Conclusions

The findings of our study lay the groundwork for future research in this area. Going forward, focus could shift to exploring the long-term effects of CRM training, particularly how it influences workplace practices and its impact on patient outcomes. There is also a need to develop more comprehensive clinical skills evaluation methods to better assess the transferability of these skills in the real-world settings. Moreover, the potential challenges unveiled in our study, such as physical exhaustion during training, must be considered when refining and designing such modules.

### Electronic supplementary material

Below is the link to the electronic supplementary material.

Supplementary Material 1

## Data Availability

The datasets used and analyzed during the current study are available from the corresponding author on reasonable request.

## Abbreviations

AKU: The Aga Khan University
CRM: Crisis Resource Management
CSACD: Collaboration and Satisfaction about Care Decisions
CTS: Clinical Teamwork Scale
FGD: Focused Group Discussion
GRS: Global Rating Scale
MBBS: Bachelor of Medicine, Bachelor of Surgery
SBAR: Situation, Background, Assessment, and Recommendation
STATA: Statistical software for data science
