# Supplementary material for: Transforming the delivery of care from “I” to “We” by developing the crisis resource management skills in pediatric interprofessional teams to handle common emergencies through simulation
Source: BMC Med Educ. 2024 Jun 11;24:649. doi: 10.1186/s12909-024-05459-2 (PMC11167930; doi:10.1186/s12909-024-05459-2)
Supplement: Supplementary file 1 — Supplementary Material 1 [file 12909_2024_5459_MOESM1_ESM.pdf]

Supplementary Material 1

Focused group discussion guide

Number of participants: _______________

Date: ___________________________ Duration: _____________

**Questions**	**Probes**
1- Have your ever been able to take part in a CRM situation? How was your experience?	Preparedness, time lapse, patient outcome, burn out level, collaboration, teamwork
2- How have you been taught about CRM skills in your individual program?	Formal training, informal training, expectations,
3- Please elaborate on your experience of being part of this project	Timing, grouping, teaching material, training, assessment, teamwork, skills development
4- Please elaborate on aspects which facilitated your learning in this project and how.	Timing, grouping, teaching material, training, assessment
5- Please elaborate on aspects which hindered your learning in this project and how.	Timing, grouping, teaching material, training, assessment
6- How do you want to see this training opportunity in future?	Interprofessional, longitudinal, blended etc
7- Please elaborate on aspects which you think this training will help you in.	Patient management, patient outcome, collaboration etc.
